# First Report of Sublingual Gland Ducts: Visualization by Dynamic MR Sialography and Its Clinical Application

**DOI:** 10.3390/jcm9113676

**Published:** 2020-11-16

**Authors:** Tatsurou Tanaka, Masafumi Oda, Nao Wakasugi-Sato, Takaaki Joujima, Yuichi Miyamura, Manabu Habu, Masaaki Kodama, Osamu Takahashi, Teppei Sago, Shinobu Matsumoto-Takeda, Ikuko Nishida, Hiroki Tsurushima, Yasushi Otani, Daigo Yoshiga, Masaaki Sasaguri, Yasuhiro Morimoto

**Affiliations:** 1 Division of Oral and Maxillofacial Radiology, Kyushu Dental University, Kitakyushu 803-8580, Japan; t-tanaka@kyu-dent.ac.jp (T.T.); r07oda@fa.kyu-dent.ac.jp (M.O.); r16wakasugi@fa.kyu-dent.ac.jp (N.W.-S.); r16jojima@fa.kyu-dent.ac.jp (T.J.); r16miyamura@fa.kyu-dent.ac.jp (Y.M.); r17matsumoto@fa.kyu-dent.ac.jp (S.M.-T.); 2 Division of Maxillofacial Surgery, Kyushu Dental University, Kitakyushu 803-8580, Japan; h-manabu@kyu-dent.ac.jp (M.H.); r07takahashi@fa.kyu-dent.ac.jp (O.T.); r13sasaguri@fa.kyu-dent.ac.jp (M.S.); 3 Department of Oral and Maxillofacial Surgery, Japan Seafarers Relief Association Moji Ekisaikai Hospital, Kyushu 801-8550, Japan; kodama@ekisaikai-moji.jp; 4 Division of Dental Anesthesiology, Kyushu Dental University, Kitakyushu 803-8580, Japan; r07sagou@fa.kyu-dent.ac.jp; 5 Division of Developmental Stomatognathic Function Science, Kyushu Dental University, Kitakyushu 803-8580, Japan; nishida@kyu-dent.ac.jp; 6 Division of Oral Medicine, Kyushu Dental University, Kitakyushu 803-8580, Japan; r17tsurushima@fa.kyu-dent.ac.jp (H.T.); r17otani@fa.kyu-dent.ac.jp (Y.O.); r11yoshiga@fa.kyu-dent.ac.jp (D.Y.)

**Keywords:** MR sialography, dynamic, sublingual gland ducts

## Abstract

This study was done to determine whether the sublingual gland ducts could be visualized and/or their function assessed by MR sialography and dynamic MR sialography and to elucidate the clinical significance of the visualization and/or evaluation of the function of sublingual gland ducts by clinical application of these techniques. In 20 adult volunteers, 19 elderly volunteers, and 7 patients with sublingual gland disease, morphological and functional evaluations were done by MR sialography and dynamic MR sialography. Next, four parameters, including the time-dependent changes (change ratio) in the maximum area of the detectable sublingual gland ducts in dynamic MR sialographic images and data were analyzed. Sublingual gland ducts could be accurately visualized in 16 adult volunteers, 12 elderly volunteers, and 5 patients. No significant differences in the four parameters in detectable duct areas of sublingual glands were found among the three groups. In one patient with a ranula, the lesion could be correctly diagnosed as a ranula by MR sialography because the mass was clearly derived from sublingual gland ducts. This is the first report of successful visualization of sublingual gland ducts. In addition, the present study suggests that MR sialography can be more useful in the diagnosis of patients with lesions of sublingual gland ducts.

## 1. Introduction

There have been many studies of clinical applications of magnetic resonance imaging (MRI) for evaluation, in addition to the evaluation of morphology, due to the higher quality of MRI technology [1,2,3,4,5,6,7,8]. As for our study, the technique of “dynamic MR sialography” was named and introduced because of its clinical usefulness in visualizing the excretion of saliva from the parotid and submandibular glands, and in evaluating the diagnosis of morphology and functions for both glands and the outcomes of treatments for Sjögren’s syndrome and xerostomia [1,9,10,11,12]. However, the visualization of the sublingual gland ducts is not considered even on MR imaging because it is difficult to visualize the very thin and short ducts, as seen in anatomy textbooks [13].

In our experience, the sublingual gland duct-like structures visualized and the possibility of visualizing the sublingual gland ducts by MR sialography needed to be elucidated.

In the present study, the sublingual gland ducts could be visualized. In addition, the clinical significance of the visualization and/or evaluation of the function of sublingual gland ducts was evaluated by clinical application of these techniques for some patients with sublingual gland diseases.

## 2. Materials and Methods

A total of 20 adult volunteers (9 males and 11 females, mean age 41.5 years, age range 18–56 years) and 19 elderly volunteers (8 males and 11 females, mean age 67.8 years, age range 60–80 years) over the age of 60 years, with no sublingual gland-related diseases, as confirmed by both a history and clinical examination, were recruited (Table 1). In addition, 7 consecutive patients (3 males and 4 females, mean age 43.4 years, age range 19–76 years) were also recruited, with 5 having inflammations of the oral floor, including the sublingual glands, and 2 with ranulas (Table 1). The image of a single side (randomly chosen) or a disease-related side of the sublingual gland ducts was used, since only single images could be acquired at one given time for functional evaluation. The total volume of the sublingual gland ducts was also analyzed using the images. Approval for the present study was obtained from the institutional review board of Kyushu Dental University (No. 20-27).

As in our previous reports, all images were acquired using a 1.5T full-body MR system (EXCELART Vantage powered by Atlas PPP; Toshiba, Tokyo, Japan) with a head coil (Atlas Head SPEEDER) to visualize the sublingual gland ducts, such as the parotid and submandibular gland ducts, according to Oda et al. [14]. T1-weighted, short tau inversion recovery (STIR), three-dimensional (3D) fast asymmetric spin-echo, and 2D-FASE images were acquired for each subject. The MRI parameters that were used are shown in Table 2. The 2D-FASE images were acquired after a single excitation with specific encoding for each echo. Fat saturation suppressed signals from subcutaneous fat.

The 3D MR sialography for sublingual gland ducts was performed as described by Oda et al. [14]. Briefly, in the same session where conventional MR studies of the sublingual glands were obtained, MR sialography was performed using 3D-FASE sequencing. In the 3D-FASE imaging, after a single excitation, images were acquired with a specific encoding for each echo. Fat saturation suppressed the signals from the subcutaneous fat. The imaging volume was centered parasagittally for the midline of the sublingual gland. In all volunteers and patients, post-processing of the MR sialographic images was performed for maximum intensity projection (MIP) reconstructions. Since 3D acquisitions can be reformatted into any required orientation, the sublingual gland ducts were identified on an initial set of axial 3D-FASE images, and oblique sagittal acquisition was used to capture the image of the parotid gland and/or submandibular gland ducts. The imaging time required for MR sialographic 3D reconstruction images using 3D-FASE sequencing was less than 5 min.

Dynamic MR sialographic images and data were acquired using the method described by Oda et al. [14]. First, 2D-FASE sequencing was repeated every 18 s of acquisition time and 12 s of interval time before and after the placement of several drops of 5% citric acid (1 mL) on the tongue, using a device similar to a syringe to acquire the dynamic MR sialography. Fat saturation was also applied for the suppression of signals from subcutaneous fat. The acquisition time of the dynamic MR sialography was about 7 min after stimulation. For the prevention of movement artifacts, head rests were used with a flat long cord with non-magnetic materials.

Each digitized image acquired by the dynamic MR sialography was linked to the Ziostation2 (Ziosoft, Tokyo, Japan). The detectable area in the parotid or submandibular gland ducts on the respective images and the time from post-stimulation to the return to the baseline state of the ducts pre-stimulation were measured using the scanner-computer analysis system. For each patient, the change in ratio of the detectable area in the sublingual gland ducts in respective images to the detectable area pre-citric acid stimulation was also analyzed. A graph demonstrated the connection between the time post-stimulation (*x*-axis) and the change ratio of the dynamic MR sialographic data (*y*-axis). We commonly used the graph to show the connection between the time, post-stimulation, and the change ratio for the standardization of volunteers and patients.

Using the graph of dynamic MR sialography, the diagnostic parameters were also analyzed as follows: (1) the maximum area of the sublingual gland ducts pre-citric acid stimulation; (2) the change ratio (change ratio = detectable area of sublingual gland ducts post-citric acid stimulation/detectable area pre-citric acid stimulation); (3) the time from the end of post-stimulation to the occurrence of the maximum area of the sublingual gland ducts; and (4) the time it took for the sublingual gland ducts to decrease from their maximum level to 50% of the pre-stimulation level.

The Mann–Whitney U test was used to examine the differences between the following: (1) the maximum area of the sublingual gland ducts between the adult and elderly volunteers; (2) the degree of difference between the maximum and minimum duct areas based on computer calculations between the adult and elderly volunteers; (3) the time from the end of citric acid stimulation to the occurrence of the maximum area of the sublingual gland ducts between the adult and elderly volunteers; and (4) the time required for the sublingual gland ducts to decrease from their maximum level to the 50% pre-citric acid stimulation level between the adult and elderly volunteers. *p* values less than 0.05 indicated a significant difference.

## 3. Results

### 3.1. Visualization of Sublingual Gland Ducts by MR Sialography

**Figure 1:**
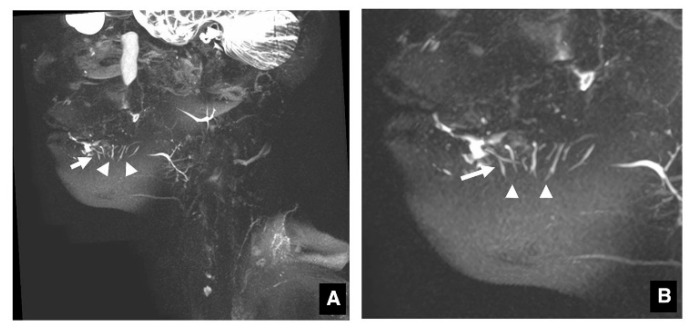
MR sialography of sublingual gland ducts. Extraglandular portions of the typical sublingual gland ducts in MR sialography ((**A**): overall image, (**B**): enlarged image of the sublingual gland area) can be identified as many bright, homogeneous, ascending linear structures (arrows) in continuity with the sublingual glands (arrowheads).

Extraglandular portions of the typical sublingual gland ducts on MR sialography could be identified as many bright, homogeneous, ascending linear structures in continuity with the sublingual glands (Figure 1)**.** The MR sialographic 3D reconstruction images of the respective angles were more easily visualized when the angle was determined manually using the mouse accompanying the MRI system. Both sublingual glands and sublingual gland ducts could be accurately visualized in 16 of the 20 adult volunteers, 12 of the 19 elderly volunteers, and 4 of the 7 patients, but only the sublingual glands were visualized in 2 adult volunteers, 4 elderly volunteers, and 1 patient (Table 3).

### 3.2. Function of Sublingual Gland Ducts Evaluated by Dynamic MR Sialography

**Figure 2:**
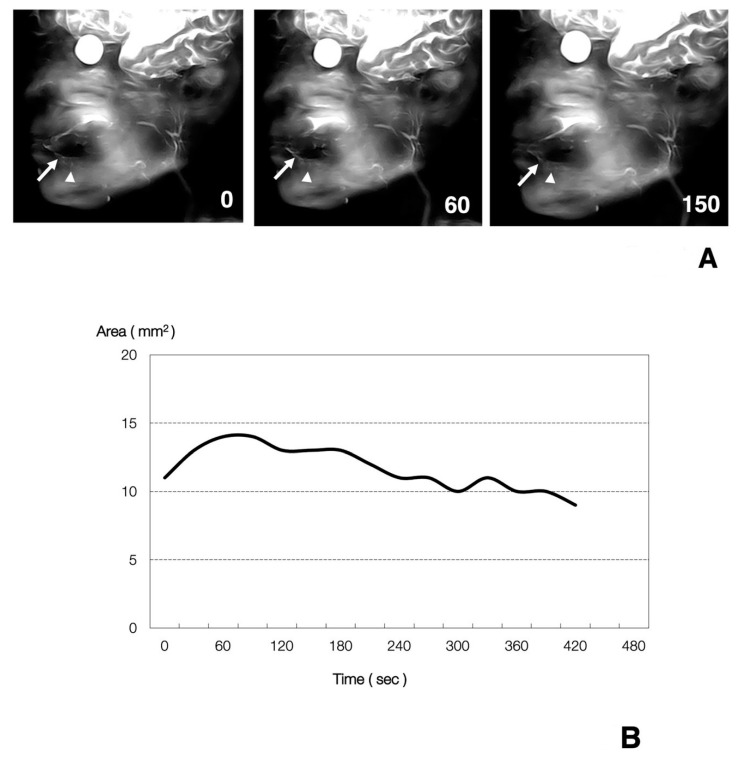
Dynamic MR sialographic images (**A**) and a graph (**B**) of sublingual gland ducts in a 26-year-old, healthy female volunteer. (**A**) The sublingual gland (arrowheads) and ducts (arrows) are gradually and slightly more clearly visualized after stimulation with citric acid for up to 60 s in a time-dependent manner. After 150 s, the many ducts became slightly clearer in a time-dependent fashion. (**B**) A graph of MR data of the sublingual gland ducts in Figure 2A demonstrates the connection between the time post-stimulation (*x*-axis) and the change ratio (*y*-axis). The area is seen at first to only increase slightly until 60 s in a time-dependent fashion. The maximum change ratio is about 1.2.

Normal dynamic MR sialography images obtained before and after citric acid stimulation are shown in Figure 2. The sublingual gland ducts were identified as many bright, homogeneous, ascending linear structures in continuity with the sublingual glands, as mentioned above (Figure 2A). The many ducts became slightly clearer in a time-dependent fashion gradually after citric acid stimulation and up to 30–60 s post-stimulation. Thereafter, the many ducts became slightly clearer in a time-dependent fashion. In the graph demonstrating the relationship between the time course post-citric acid stimulation and the change ratio of the detectable area in the sublingual gland ducts, the area was seen at first to only increase slightly to 30 s in a time-dependent fashion (Figure 2B).

The volunteers’ data are summarized in Table 4. Before citric acid stimulation, the maximum area of the sublingual gland ducts was 10.0 mm^2^ (mean ± SD = 10.0 ± 4.6 mm^2^) in the 16 adult volunteers, 9.0 mm^2^ (mean ± SD = 9.0 ± 3.4 mm^2^) in the 3 elderly volunteers, and 10.2 mm^2^ (mean ± SD = 10.2 ± 5.5 mm^2^) in the 5 patients (adult vs. elderly: *p* = 0.21, adult vs. patients: *p* = 0.46, elderly vs. patients: *p* = 0.92; Mann–Whitney U test). After citric acid stimulation, the maximum area of the parotid gland duct was 13.2 mm^2^ (mean ± SD = 13.2 ± 5.3 mm^2^) in the 16 adult volunteers, 10.7 mm^2^ (mean ± SD = 10.7 ± 4.4 mm^2^) in the 12 elderly volunteers, and 11.0 mm^2^ (mean ± SD = 11.0 ± 5.4 mm^2^) in the 5 patients (adult vs. elderly: *p* = 0.53, adult vs. patients: *p* = 0.94, elderly vs. patients: *p* = 0.67; Mann–Whitney U test).

After stimulation, the time of occurrence of the maximum duct area varied from 30 s to 180 s in all subjects (mean ± SD = 62 ± 28 s in the 16 adult volunteers, mean ± SD = 63 ± 26 s in the 12 elderly volunteers, and mean ± SD = 54 ± 13 s in the 5 patients); (adult vs. elderly: *p* = 0.92, adult vs. patients: *p* = 0.40, elderly vs. patients: *p* = 0.39; Mann–Whitney U test). The time it took for the detectable duct area to return to almost 50% of its former area was about 115 s in all subjects (mean ± SD = 110 ± 39 s in the 12 adult volunteers, mean ± SD = 117 ± 57 s in the 12 elderly volunteers, and mean ± SD = 114 ± 25 s in the 5 patients); (adult vs. elderly: *p* = 0.76, adult vs. patients: *p* = 0.82, elderly vs. patients: *p* = 0.89; Mann–Whitney U test). No significant differences in the four parameters, including the change ratio (adult vs. elderly: *p* = 0.24, adult vs. patients: *p* = 0.19, elderly vs. patients: *p* = 0.26; Mann–Whitney U test) in the detectable duct area of the sublingual glands were found among the three groups (Table 4).

### 3.3. Clinical Application of MR Sialography for Patients with Sublingual Gland Diseases

**Figure 3:**
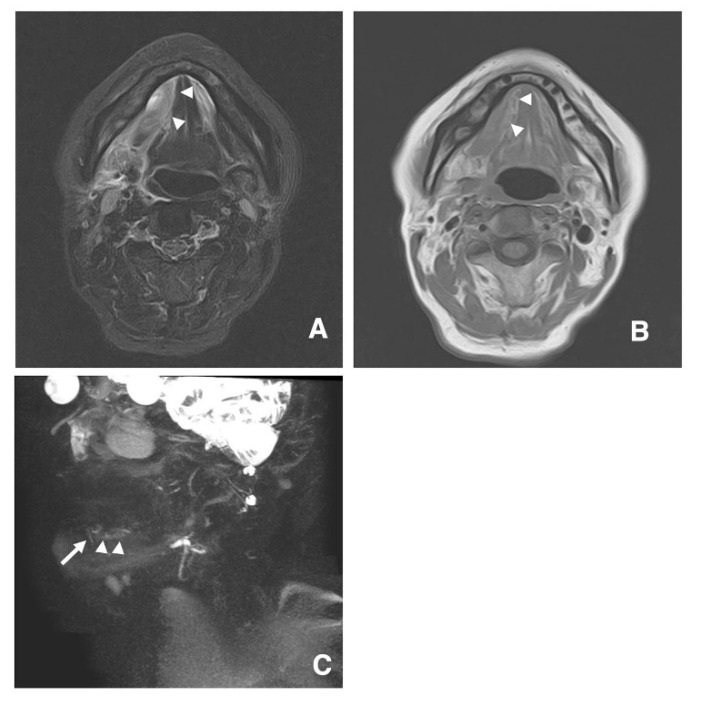
STIR (**A**), T1-weighted images (**B**), and MR sialography (**C**) of a 76-year-old man with inflammation of the right oral floor. The disappearance (arrows) of many sublingual gland ducts in continuity with the sublingual glands is visualized using MR sialography.

In a 76-year-old man with inflammation of the right oral floor, many sublingual gland ducts continued with the sublingual glands in STIR, T1-weighted images, and MR sialography (Figure 3A–C).

**Figure 4:**
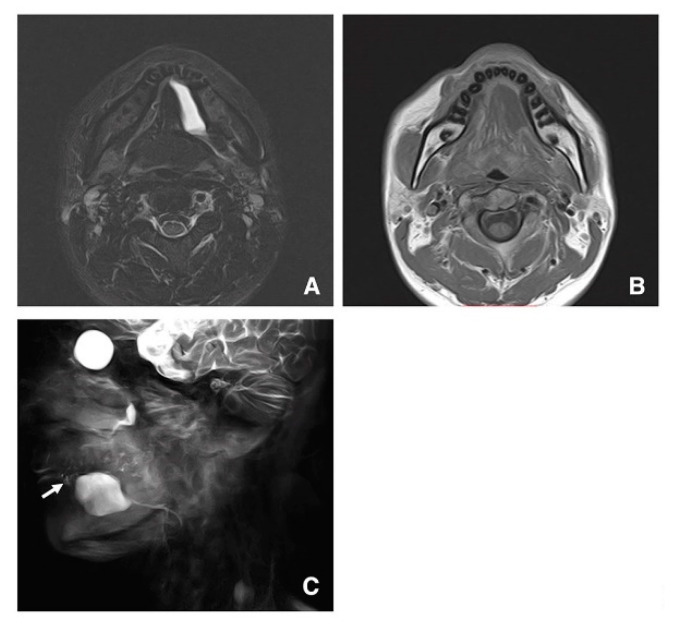
STIR (**A**), T1-weighted image (**B**), and MR sialography (**C**) of a 30-year-old woman with a ranula on the left. The mass lesion is seen in continuity with the sublingual glands in STIR (**A**) and T1-weighted images (**B**) and is diagnosed as a ranula. The mass is derived from one of many sublingual gland ducts (arrow) (**C**).

In a 30-year-old woman with a ranula on the left, the mass lesion was detected in continuity with the sublingual glands in STIR and T1-weighted images and was thus diagnosed as a ranula (Figure 4A,B). In addition, the mass was derived from one of the many sublingual gland ducts in images obtained using MR sialography (Figure 4C).

## 4. Discussion

The most interesting result of the present study is that it is the first to show the imaging characteristics of the sublingual gland ducts obtained by 3D MR sialography. It is otherwise difficult to visualize the very thin and very short ducts, with a diameter and length of only 1 mm, as shown in anatomy textbooks [13]. This is a very significant first success in salivary gland imaging. The extraglandular portions of the typical sublingual gland ducts appeared as many bright, homogeneous, ascending linear structures in continuity with the sublingual glands. The figures of the sublingual gland ducts obtained by MR sialography were the same as those in a textbook of oral anatomy [13]. Therefore, the images of the structures could be confirmed to be sublingual gland ducts using MR sialography. However, the sublingual gland duct could not be detected in all subjects as the detection rate was about 57.1%. One possible explanation is that the sublingual gland ducts are so thin and short that they cannot be visualized in all subjects using MR sialography.

So far, the reason the sublingual gland ducts, with their thin and short size, have not been visualized before, even in MR images, is that visualization has been considered technically impossible. In addition, it was thought that there was little clinical significance in the visualization of sublingual gland ducts. However, these very sublingual gland duct-like structures were visualized in the MR sialography of patients with submandibular and/or parotid gland-related diseases. Therefore, we planned the present study of the visualization of sublingual gland ducts using MR sialography.

One other interesting result of the present study is that the visualization of sublingual gland ducts indicates the clinical significance of sublingual gland-related diseases. The visualization of sublingual gland ducts concretely demonstrated a mass derived from one of many sublingual gland ducts. Based on the imaging, the mass should have been diagnosed as a ranula. At the same time, the disappearance of many sublingual gland ducts in continuity with sublingual glands was visualized using MR sialography in patients with inflammation on the right. We would like to elucidate the clinical significance of the MR sialography of sublingual gland ducts for many kinds of diseases in the oral floor, including sublingual gland-related diseases.

One reason for this first success in the visualization of sublingual gland ducts is that MR systems, 3D computer vision, and image processing techniques have been fast advancing due to the growing computational power of current computer systems. Rapid advances in 3D data acquisition and post-processing technologies are expanding the potential applications of 3D displays. In the 1.5T full-body MR system (EXCELART Vantage powered by Atlas PPP; Toshiba, Tokyo, Japan) with a head coil (Atlas Head SPEEDER), 3D-FASE was used for the sequencing of MR data sets through the acquisition of MR sialographic 3D reconstruction images, since this method was most likely to provide good resolution in a short period of time, as in our previous report [15]. That both types of images had good resolution using 3D-FASE sequencing may have been due to the section thickness being as little as 1 mm, despite the short acquisition time [16]. The section thickness was based on the advantage that 3D-FASE sequencing could excite a three-dimensional sample [17]. Thin slices produce images with good resolution while minimizing interference from partial volume effects and the formation of artifacts in the MR data and workstation. In addition, an adequate Fourier transform may be applied to 3D-FASE sequencing to produce high-resolution images [17]. Another advantage is that an image can be acquired using additional excitation, without conducting an additional imaging session, in cases when using a single excitation would not produce a satisfactory image. These unusual and useful characteristics of 3D-FASE sequencing allow for the avoidance of unnecessary exposure of patients to the RF pulse and an unnecessarily long acquisition time.

To our regret, little alteration of the dynamic curve was seen by dynamic MR sialography of sublingual gland ducts. This can be considered physiologically correct and reasonable [18,19]. Physiologically stimulated saliva production is the main role of the parotid glands [18,19]. The saliva flow rate of the parotid glands increases more quickly than that of the submandibular glands during citric acid stimulation [20,21,22]. Resting saliva is produced as the main role of the submandibular glands [18,19]. The salivary flow rate in the parotid gland during stimulation is twice as high as that in the rest phase, but less of an increase is found in the submandibular gland [23,24]. The main role of sublingual glands, however, is to keep the oral mucosa moist, but not to maintain resting and stimulated saliva flow. Therefore, little alteration of the dynamic curve via dynamic MR sialography of sublingual gland ducts was seen. We are now planning to elucidate the clinical significance of dynamic MR sialography of the sublingual gland ducts through its clinical application to many kinds of diseases in the oral floor, including sublingual gland-related diseases.

One possible limitation of the present data is the small sample size. The variables of race and sex could not be studied in this study sample. In addition, only a few clinical applications were examined. Therefore, further investigation is required. In the present study, there was little witness of movement artifacts by the volunteers, but we predicted that patients would move in MR examinations, despite our system preventing movement artifacts. Moreover, we paid attention to the possibility of visualizing sublingual gland ducts using dynamic MR sialography and its clinical application in the present study. Therefore, we could not elucidate the classification of the drainage of the sublingual glands in Bartholin’s ducts and/or the duct of Rivinus. At the next stage, we should try to classify their drainage patterns. At the same time, we should try to elucidate how the presence of Bartholin’s ducts may be related to ranula formation as the next stage. We added the sentence mentioned above in the revised manuscript.

## 5. Conclusions

MR sialography allows for the evaluation of function and morphology of the sublingual gland ducts. This technique appears to have many possible applications in the dental, medical, and biological fields.

## Figures and Tables

**Table 1:** Subjects.

	Male			Female		
	Number	Age (Mean ± SD)	Age Range	Number	Age (Mean ± SD)	Age Range
Adult volunteers	9	46.5 ± 8.7	29–55	11	40.3 ± 12.9	18–56
Elderly volunteers	8	68.1 ± 5.5	61–79	11	67. 5 ± 6.5	60–80
Patients	3	37.3 ± 15.4	27–55	4	48.0 ± 24.3	19–76

**Table 2:** Imaging parameters of each sequence.

	Sequence			
	STIR	T1WI	2D-FASE	3D-FASE
TR (ms)	4700	820	6000	3.2
TE (ms)	75	15	250	1.6
Flip angle (°)	90	90	90	45
FOV (mm)	200 × 200	200 × 200	200 × 200	200 × 200
Section thickness (mm)	6	6	30–60	1.8
Matrix (pixels)	224 × 320	224 × 320	224 × 320	120 × 96
Acquisition time (min: s)	3:30	3:30	0:18 (12hase)	4:30–5:30

TR: Repetition time, TE: Echo time, FOV: Field of view, STIR: Short T1 inversion recovery, T1WI: T1-weighted image, 2D-FASE: 2-dimensional fast asymmetric spin-echo, 3D-FASE: 3-dimensional fast asymmetric spin-echo.

**Table 3:** Summary of visualization of sublingual gland ducts by MR sialography.

	Sublingual Glands and Ducts Visualized	Only Sublingual Glands Visualized
Adult volunteers (*n* = 20)	16	2
Elderly volunteers (*n* = 19)	12	4
Patients (*n* = 7)	5	1

**Table 4:** Summary of physical and dynamic MR sialographic data.

	Area (mm^2^)			Period to Occurrence of Maximum Area (s)	Period to Return to Its Pre-Citric Acid Stimulation 50% Level (s)
	Before Citric Acid Stimulation	After Citric Acid Stimulation	Change Ratio		
Adult volunteers (*n* = 16)	10.0 ± 4.6	13.2 ± 5.3	1.3 ± 1.1	62 ± 28	110 ± 39
Elderly volunteers (*n* = 12)	9.0 ± 3.4	10.7 ± 4.4	1.2 ± 1.3	63 ± 26	117 ± 57
Patients (*n* = 5)	10.2 ± 5.5	11.0 ± 5.4	1.1 ± 1.0	54 ± 13	114 ± 25
